# Densification Mechanism of Soft Magnetic Composites Using Ultrasonic Compaction for Motors in EV Platforms

**DOI:** 10.3390/ma12050824

**Published:** 2019-03-11

**Authors:** Myeong-Hwan Hwang, Hae-Sol Lee, Jong-Ho Han, Dong-Hyun Kim, Hyun-Rok Cha

**Affiliations:** 1 EV Components & Materials R&D Group, Korea Institute of Industrial Technology, Gwangju 61012, Korea; han9215@kitech.re.kr (M.-H.H.); eddylee12@kitech.re.kr (H.-S.L.); jonghohan@kitech.re.kr (J.-H.H.); bjiuh2608@kitech.re.kr (D.-H.K.); 2 Department of Electrical Engineering, Chonnam National University, Gwangju 61186, Korea; 3 Robotics and Virtual Engineering, Korea University of Science and Technology, Daejeon 34113, Korea

**Keywords:** ultrasonic, compaction mechanism, internal friction reduction, SMCs (soft magnetic composites)

## Abstract

In this paper, the densification mechanism of ultrasonic compaction was analyzed using a force balance model. Ultrasonic compaction is quite a promising way to solve the lower mechanical property problem of green compact in the compaction process, although it has some obstacles to overcome for its various applications. Our model proposes that the resultant density is achieved as the applied and resistance forces reach the equilibrium state. Based on the proposed model, the ultrasonic compaction increases the density of green compact by reducing the internal friction between the powder and compaction die, as well as the internal friction among particles themselves. It was also found that during the powder compaction, the ultrasonic vibration mostly contributes to slipping and the rearrangement of the particles.

## 1. Introduction

**Figure 1:**
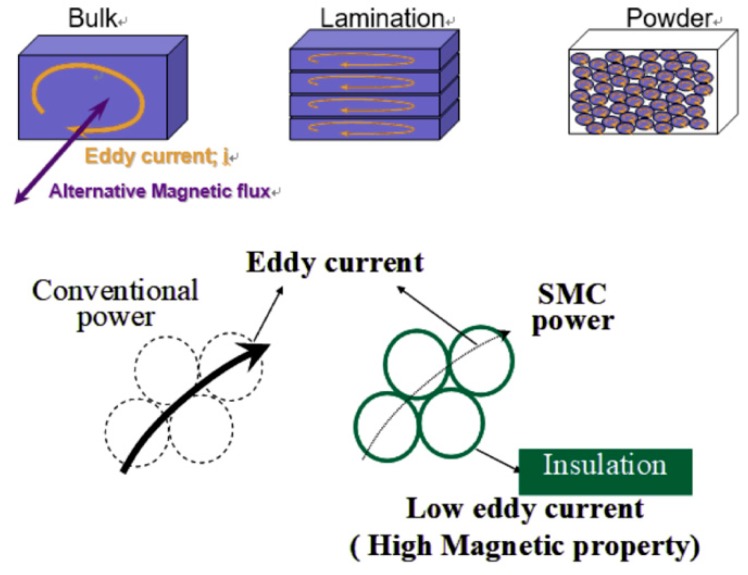
The occurring mechanism of the eddy current loss.

During recent years, interest in the study of the soft magnetic composites (SMCs) has increased in the magnetic and electric industries. SMC materials are basically pure iron powder particles coated with very thin electrically insulating layers. As Figure 1 shows, the insulating layer restricts the eddy current, which is generated by an alternating magnetic field to mainly travel within each individual particle. Due to eddy current restriction, SMC materials provide new opportunities to design compact, light, and cost-efficient solutions for high-performance motors [1,2,3,4].

**Figure 2:**
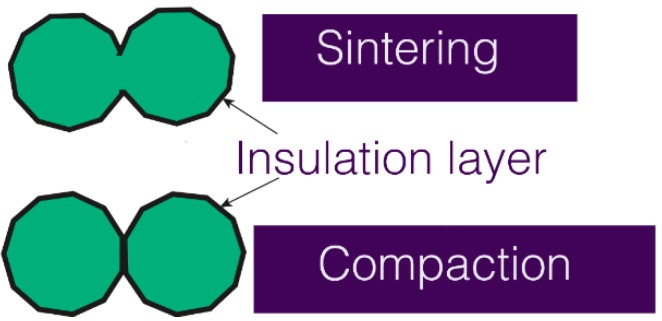
Heat applied during sintering causes defects in the insulation layer.

In conventional powder metallurgy processing, the parts are sintered after compaction. Sintering is a high-temperature heat treatment which develops the bonds between the powder particles and enhances their mechanical performance. However, SMC powders cannot be sintered, because they reduce the eddy current. If the magnetic flux changes in the magnetic substance, an electromotive force is generated—a swirl of current flows through the magnetron. This is called an eddy current, which consumes the mechanical energy of the driving part in Joule heating, thus reducing efficiency. To reduce this, the particles should not be bonded to each other, as shown in Figure 1. The temperatures involved would cause the insulation coating to break down, giving a poor electrical performance as the particles would bond to each other as shown in Figure 2 [5,6]. Therefore, in order to form an SMC, only the compaction method is applied.

It is well known that high-frequency vibrations, such as ultrasonic vibration, reduce particle friction [7,8,9]. Researchers proposed the usage of ultrasonic vibration during the powder compaction process to increase the density and reduce the internal friction without the increase in compaction pressure. J. Tsujino et al. reported the ultrasonic vibration press of copper and piezoelectric ceramic powder in a vacuum condition [10,11], and O.L. Khasanov et al. reported the ultrasonic compaction of ceramic nano-powder [12]. Both studies made the following conclusions—the ultrasonic compaction during the ceramic nano-powder compaction (a) lowered lateral pressure to a greater degree in a low compaction pressure condition, (b) more effectively decreased an elastic after-effect in a high-pressure environment, and (c) improved the green compact quality [13,14,15]. 

According to the published literature, one of the effective methods for powder compaction is ultrasonic-assisted compaction under a pressure of 250 MPa, which may potentially be applicable to massive powder metallurgy manufacture processes [16].

However, the ultrasonic compaction method faces a problem that limits its wider application. Due to an unknown mechanism, the high densification phenomenon is generally accepted without clear explanations. The aim of this research, therefore, is to investigate the relationship between low internal friction forces and the high densification of powders using a force balance model. We expect that our proposed model will contribute to the wider usage of ultrasonic compaction and its successful industrialization.

In this paper, the reduction in friction and the improvement in resultant density are studied as functions of compression pressure, vibration amplitude/frequency, and input power. Through this experiment, fundamental information such as the densification mechanism under ultrasonic action and the optimum design parameter for high densification is determined. It is found that the input power needed to drive the ultrasonic vibration system is the governing parameter for obtaining the high density results of an SMC specimen.

## 2. Materials and Methods

**Figure 3:**
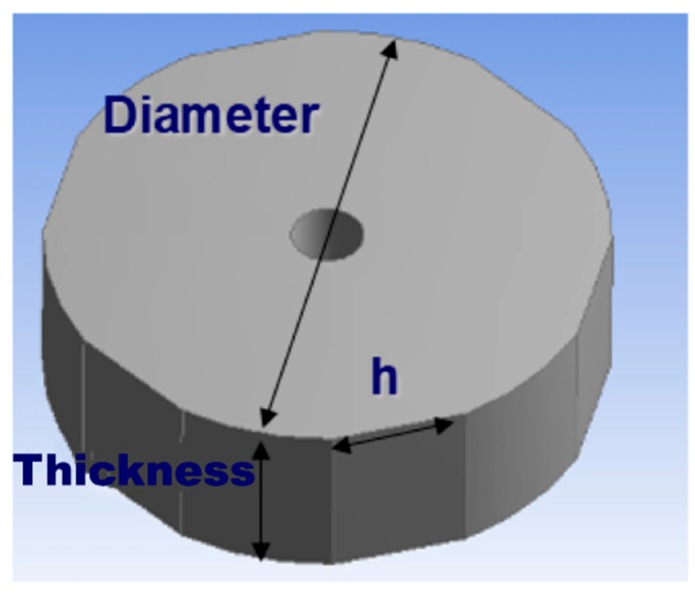
Design parameter for ultrasonic compaction.

The shape and dimensions of the die were set, as shown in Figure 3; the thickness was 50 mm, the outer diameter was 160 mm, the inner diameter was 20 mm, and the flattening curve was 40 mm for use at 19.8 kHz. 

The ultrasonic compaction variables are displayed in Table 1. The material used for this research was limited to SMCs termed SMC550 and SMC500. The coefficient of the relation equation of determining density of SMCs was firstly taken. Table 2 shows the coefficient of the equation for determining the density of SMCs without ultrasonic action. Through Table 1 and Table 2, the resultant density with ultrasonic action may consist of two portions, as shown in Equations (1) and (2); one is the density increase by the ultrasonic effect and the other is the conventional compaction effect, without the ultrasonic effect.

(1) ρ=f(u, F, A, P, t, f, fr, st, si, lu, po, mo)

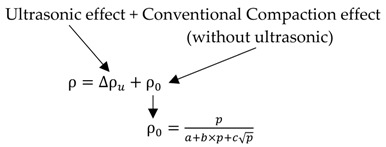
(2)

**Figure 4:**
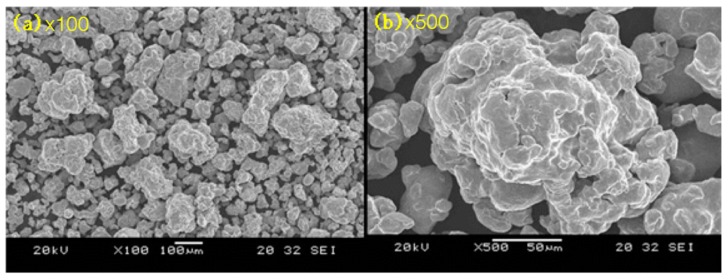
Optical microscope observations of SMC500.

To observe the voids or porosity among the particles, a microscopic analysis of the SMC powders was needed. In Figure 4, the SEM (Scanning Electron Microscope) pictures of SMC500 are shown, which were used for the analysis. 

**Figure 5:**
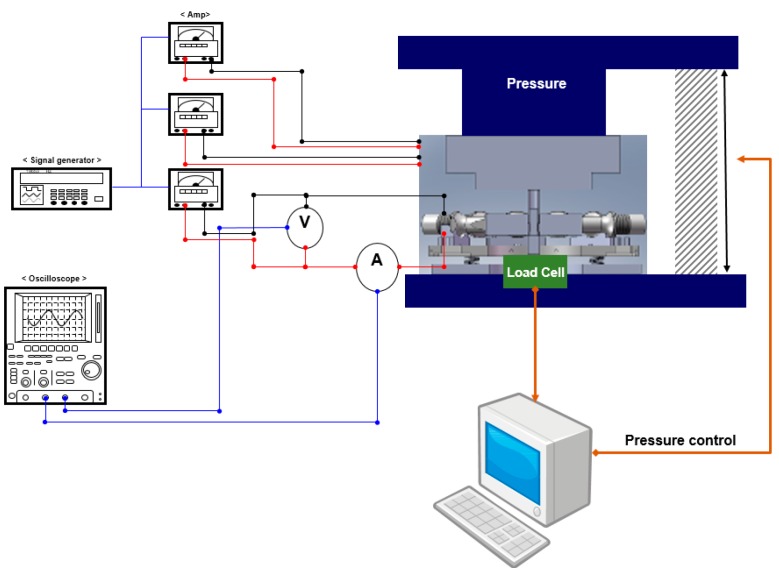
Full configuration of experiment.

The equipment installed and its setup for the experiment are shown in Figure 5, and were also published in our previous work [16]. In this research, a Universal Test Machine (UH-F500) (Shimadzu, Kyoto, Japan) was used to apply the pressure. The ultrasonic wave was produced by a signal generator (WF1943A) (NF corporation, Yokohama, Japan) and enlarged through a high-power amplifier (HSA4101) (NF corporation, Yokohama, Japan). The amplified wave was applied to each vibrator. The input current and voltage were measured by CT (Current Transformer) and PT (Potential Transformer) at the point where the ultrasonic transducer was connected and the values were displayed using an oscilloscope. Each amplifier generated a maximum voltage of 200 V and a maximum current of 1A. Also, in order to consider the effect of the driving frequency on the densification of SMCs, the change in density with different driving frequencies was measured. Here, vibration compacting die was designed for operating at 20 kHz half wavelength longitudinal vibration and the press frame had a hydraulic static compacting pressure source. The experiment was conducted for 3 seconds, so as not to destroy the insulation layer due to the temperature increase.

**Figure 6:**
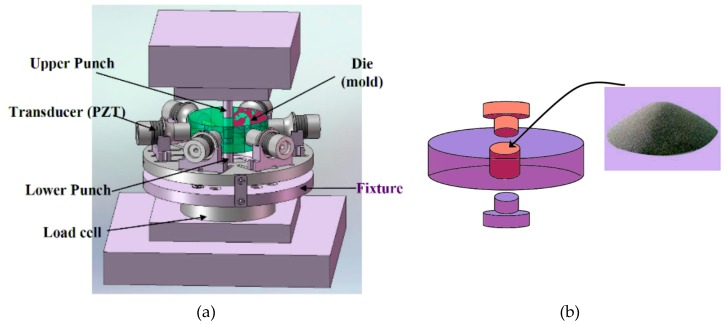
Ultrasonic vibration compaction system: (**a**) compaction system diagram, (**b**) illustration of basic operation concept.

The ultrasonic compaction system was set up as shown in Figure 6. A soft magnetic composite (SMC) was chosen among various types of powder. The SMC has a very high potential for practical use due to its outstanding magnetic characteristics, but its compact density cannot increase beyond a certain level. Hence, its mechanical strength is limited up to a certain point using a conventional compaction method. If the mechanical strength of the SMC could be improved through an ultrasonic compaction process, then more advanced materials that are made of SMCs could be produced. For this reason, the SMC was chosen to be tested among other powders.

**Figure 7:**
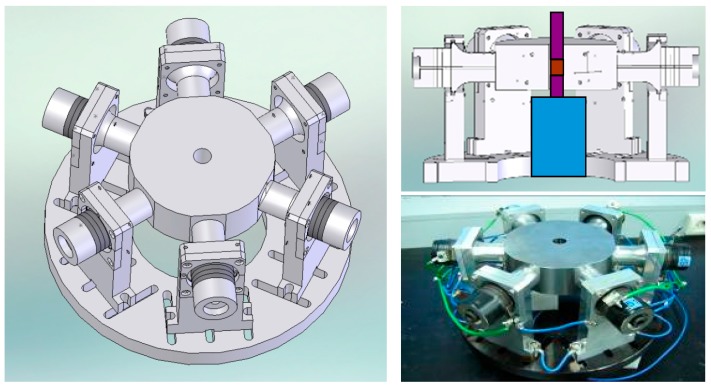
Assembled ultrasonic compaction mold system with supporting fixture.

Figure 7 shows the assembled ultrasonic compaction mold system with the supporting fixture. A total of six ultrasonic transducers were connected to the die and arranged in a radial direction. From the cross-sectional view (upper right picture in Figure 7), there was a hole where the specimen was compacted and structured. In the lower right picture of Figure 7, the connection method between the ultrasonic transducer and the fixture was also described. In order to prevent the production of an electrical shock, the ground line (green cable) was set up at the die and fixture opposite each other. 

**Figure 8:**
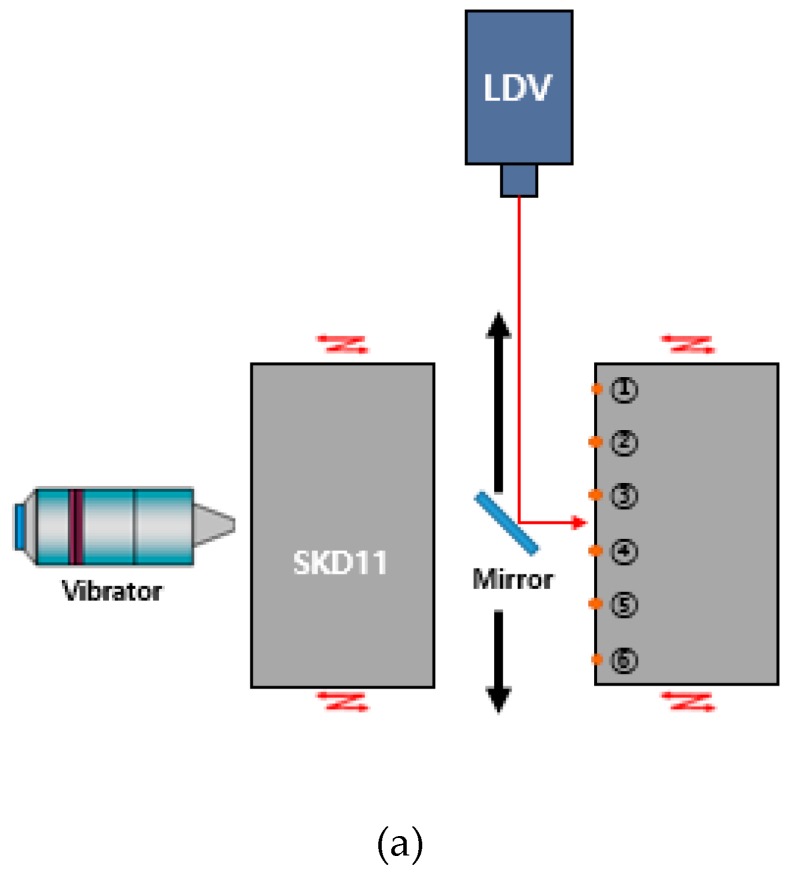

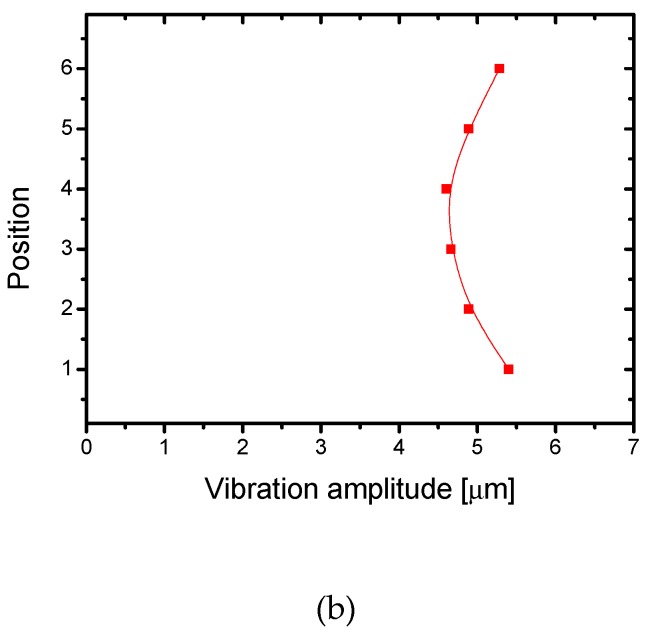
Vibration distribution of the hole where the SMC is compacted: (**a**) measurement setup; (**b**) vibration distribution according to six positions.

Figure 8 shows the measured results of the vibration distribution of the interior area of a hole where the SMC was compacted. It was measured using a mirror because there was no margin in the interior area of the hole. The measured vibration amplitude distribution agreed well with the FEM (Finite Element Method) simulation results. The maximum vibration points were ① and ⑥, and the minimum vibration points were ③ and ④. However, the deviation of the vibration amplitude between the maximum and minimum points was almost the same at each point with a deviation of 1.5 μm. The vibration amplitude of position No.1 was 5.5 μm.

**Figure 9:**
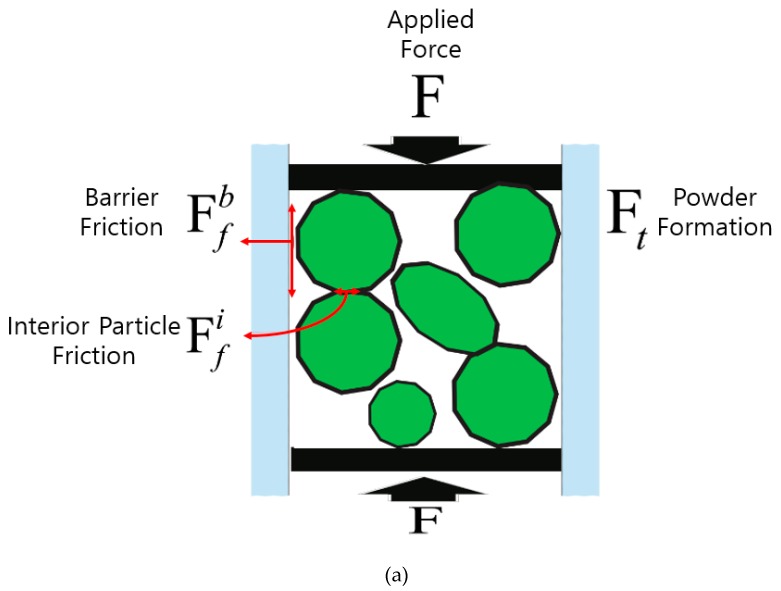

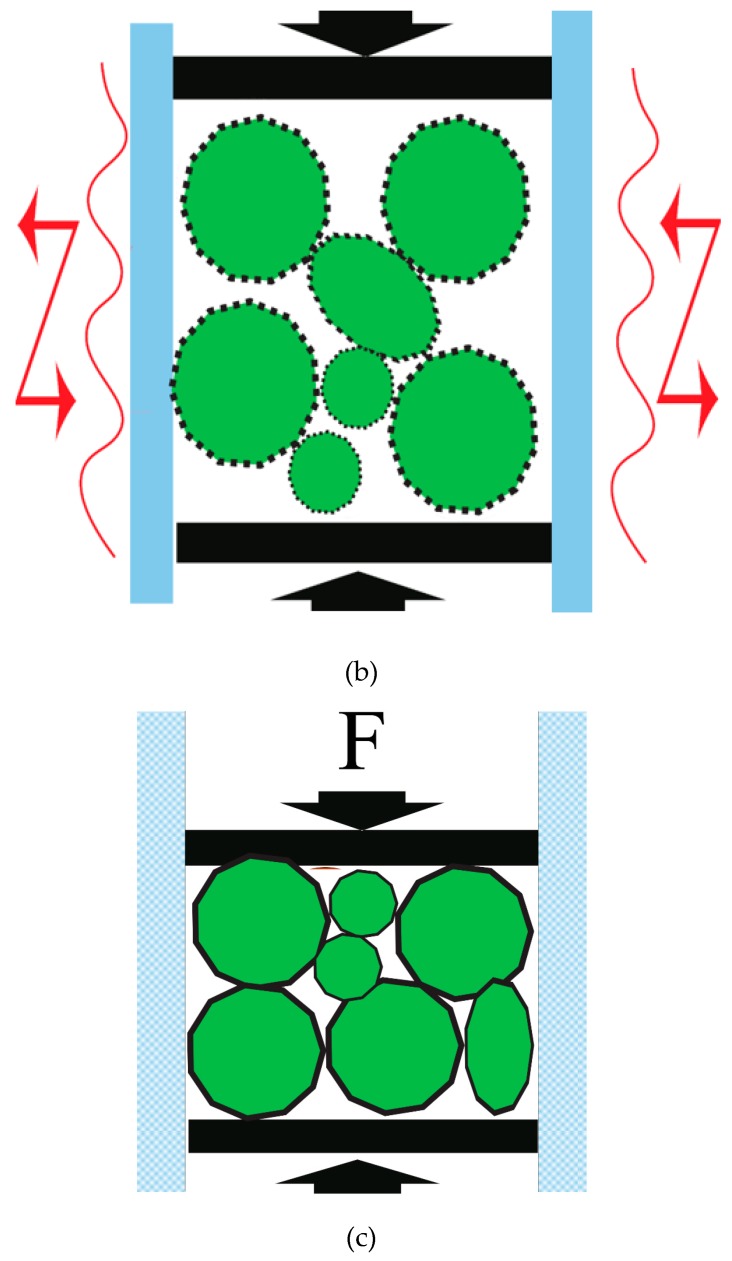
Procedure of ultrasonic compaction: (**a**) stage 1—only pressure; (**b**) stage 2—pressure + ultrasonic, (**c**) final stage (after densification).

As the ultrasonic compaction is taking place, the proposed system is expected to undergo densification, as described below. In the first stage, pressure is applied to the SMC sample in the die where the density is determined by the equilibrium force, as expressed in Equation (3). The applied force contributes to the densification of the SMC as shown in Figure 9a. 

The friction between the die wall and powders as well as between the particles themselves prevent the further densification of the SMC. Densification is also affected by the formation force. Therefore, the resultant density is achieved when the applied and resistance force reach an equilibrium state.

(3) $$\sum F=\sum \mu_{b}F_{f}^{b}+\sum \mu_{i}F_{f}^{i}+\sum F_{t}$$

where ∑F: Applied force; $\sum \mu_{b}F_{f}^{b}$: Barrier friction force; $\sum \mu_{i}F_{f}^{i}$: Inter-particle friction; $\sum F_{t}$: Formation force. $\mu_{b}$: Friction coefficient between barrier and powder; $\mu_{i}$: Friction coefficient among particles themselves; 

In the second stage, the ultrasonic vibration is applied to the die through BLT (Bolt Clamped Langevin Type) -type ultrasonic transducers while the pressure is applied, as shown in the Figure 9b. Ultrasonic vibration is an effective way to reduce the internal friction [11], because it has a small amplitude and high frequency. Vibration of the die wall, therefore, is expected to reduce the internal friction between the die wall and particles, as well as the inter-particle friction in a system. The SMC powder then rearranges itself to go into empty spots due to the lowered internal friction, and the further densification of SMC is achieved as shown in Figure 9c.

**Figure 10:**
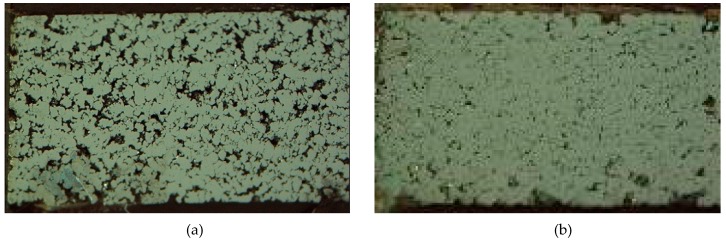
Optical microscope observation of compacted material with and without ultrasonic vibration: (**a**) conventional compaction; (**b**) ultrasonic compaction.

To observe the voids or porosity among the particles after compaction, a microscopic analysis was carried out. In Figure 10, the microstructures of compacts with and without ultrasonic vibration are shown with a compressive pressure of 300 Mpa. Because of the low pressure applied, the experiment was in the “repacking” stage. As shown, there was higher porosity exhibited after the conventional compaction method than after ultrasonic vibration. Even with the same compressive pressure, the density increment by the reduction of porosity between the particles was clearly observed in the microstructure.

## 3. Analysis of Densification Mechanism under Ultrasonic Compaction

### 3.1. Conventional Compaction Mechanism

**Figure 11:**
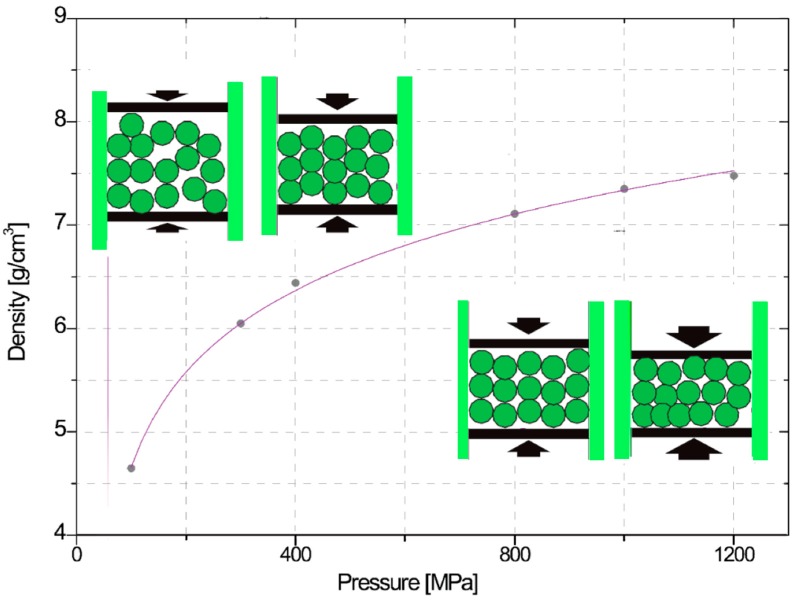
Conventional densification mechanisms under various pressure conditions.

**Figure 12:**
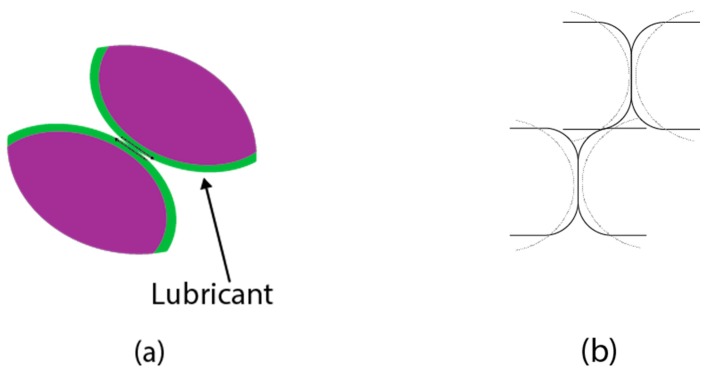
The contributing factors raising the densification: (**a**) slipping between particles during the repacking step; (**b**) deformation of the particles.

The material used for this experiment was SMC500, and for the lubricant, Kenolube was used. This is a type of organic lubricant and a proprietary lubricant, containing 2% Zn. Kenolube is sensitive to high temperatures and mostly high ambient humidity, which promotes the formation of lumps and negatively impacts the flow behavior. A general mechanism of conventional compaction is shown in Figure 11. During the first step of compaction, particles slip among themselves under pressure, and rearrange their positions without the deformation of the powder. This step is called ‘repacking’ in which the density is raised. In the second step, particles from the powder start to deform at high pressures of over 400 MPa, and this step in which the density increases is called ‘deformation’ [15]. As shown in Figure 11, there is no clear distinction between the repacking and the deformation steps. However, repacking generally occurs at a low pressure and deformation occurs at a high pressure. The dominant contributing factor raising the densification is illustrated in Figure 8, showing slipping and rearrangement between the particles in the repacking step and the deformation step under high pressure [17,18]. In Figure 12a, the blue outline indicates the lubricant.

### 3.2. Density Comparison with Ultrasonic and Non-Ultrasonic Effects under Different Compacting Pressures

**Figure 13:**
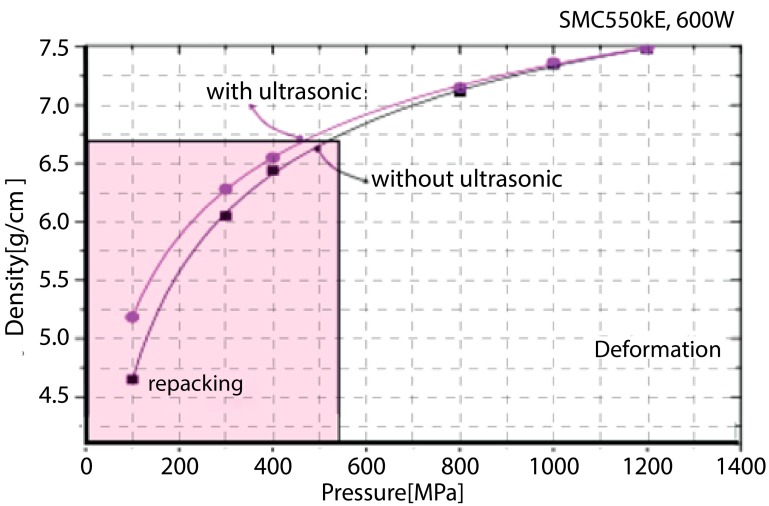
Ultrasonic vibration effect on density during compaction under different pressure conditions.

The density of the SMC test sample was measured under various pressure conditions from 100 to 1200 MPa. Figure 13 shows the density profile of the SMC sample when it was compacted with and without ultrasonic vibration during the compaction process. The SMC specimen with ultrasonic vibration showed a higher density profile than that of the SMC specimen without ultrasonic vibration. By comparing the densities between two such cases, the difference in density was larger in the repacking region than in the deformation region. From this experiment, it can be inferred that ultrasonic vibration during the compaction process mostly contributes to the slipping and rearrangement of the particles, since repacking dominantly occurs in the low-pressure range, as discussed in Section 3.1. 

### 3.3. Analysis of Repacking Effect on the SMC Density

**Figure 14:**
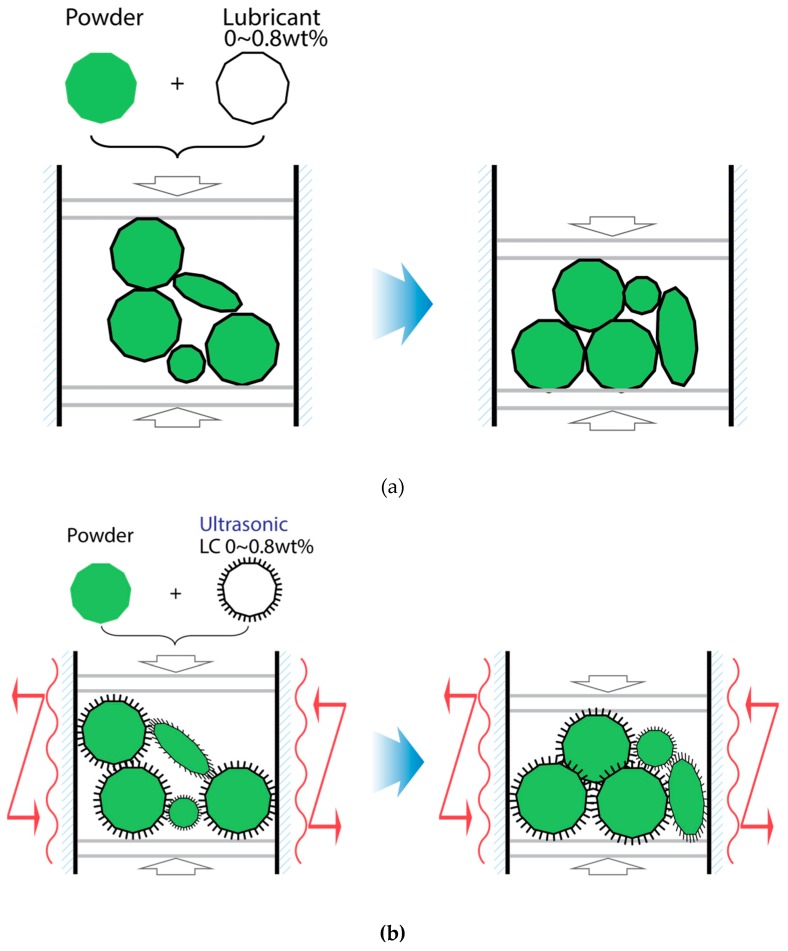
Illustration of the repacking mechanism with different amounts of lubricant usage: (**a**) compaction with lubricant usage only; (**b**) compaction with both lubricant and ultrasonic usage.

**Figure 15:**
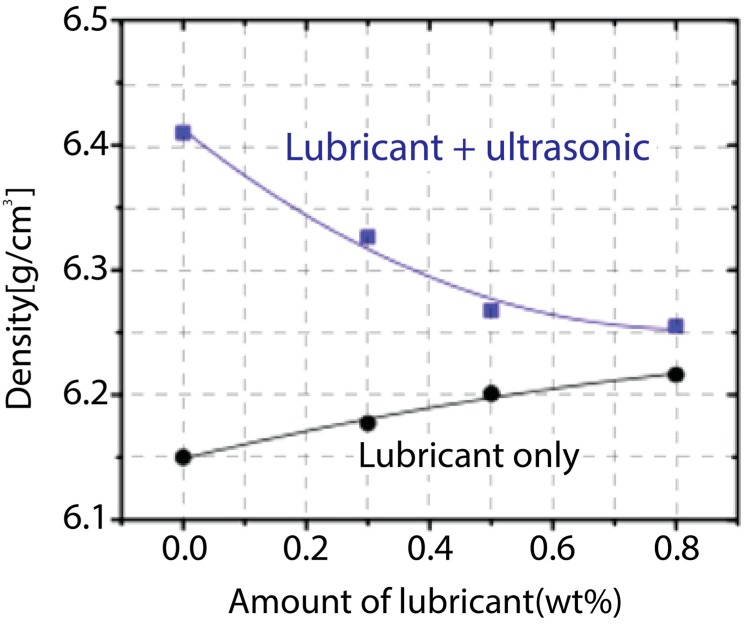
Density comparison between ultrasonic and non-ultrasonic effects under different lubrication conditions.

In order to verify the density improvement caused by the repacking step described in the previous section, powder and lubricant mixtures with a mixing ratio from 0 to 0.8 wt% lubricant were made. Lubricant is used in this experiment because it enhances the slipping of the powders during the compaction process. To compare the results from a lubricant experiment, an ultrasonic vibration, which was experimentally shown to enhance the slipping and rearrangement of particles in Figure 13, was applied to each SMC test sample. The experimental concept, which is based on a force balance model, is shown in Figure 14. Figure 14a depicts the conventional powder compaction procedures only with lubrication whereas Figure 14b shows the densification procedures with a combination of ultrasonic compaction and lubrication. At a constant pressure of 300 MPa, the density of the compacted powder was measured in both cases. The amounts of lubricant used varied from 0.0 to 0.8 wt% and the results are shown in Figure 15. 

The experimental result of the density with ultrasonic vibration is exhibited in Figure 15. As shown, when ultrasonic vibration was applied, the effect on the slipping of the powders and consequent result in the increase of density was very significant, even when the powder was not lubricated. The density of the SMC and lubricant mixture without ultrasonic effects is plotted as a black curve, and the density of the SMC and lubricant mixture with ultrasonic effects is shown as a blue curve. It is interesting to note that both black and blue curves tend to reach around the same value as an increase in lubricant was added. The lubricant plays a role in decreasing internal friction in the compaction process, as discussed in previous sections. Since both curves reach around the same value, it is experimentally proven that ultrasonic effects also play the same role as the lubricant in the compaction process, which is to decrease frictional forces. 

Although both curves direct towards the same level, the blue and black curves behave differently. The black curve increases as more lubricant is added because the SMC sample can rearrange and go easily into to the empty spots in the system due to the lowered frictional forces among the particles themselves. This rearrangement of the SMC sample eventually increases the densification of tested particles because many empty spots can be filled up with rearranged SMC particles, and thus the sample can become more compacted. As more lubricant was added, the blue line showed a downtrend. Even though the internal friction decreased as more lubricant was added, the thickness of the lubricant coating around the SMC sample increased significantly, thus cancelling out the slipping and rearrangement effects. The rearrangement of the SMC sample is not as easy as in the previous case because fewer SMC particles can go into empty spots due to their thick lubricant coating. Thickening the coating around the particles was a more dominant factor in this case, thus the blue curve decreased as more lubricant was added. In the end, the density increased about 5~10% with a high-power ultrasonic compaction system compared that of the conventional compaction system.

## 4. Conclusions

There have been many cases reported in academic societies indicating that ultrasonic compaction contributes to a higher densification of given samples. Ultrasonic compaction technology has received great attention recently since the low mechanical strength of given samples can be improved when it is used to develop the motor core. It is important to note, however, that the reasoning for this phenomenon and the densification mechanism of an ultrasonic compaction has not been reported yet. Due to this unknown mechanism, quantitative analysis of ultrasonic compaction could be done with limitations.

Based on the proposed force balance model, the experiments were designed to test and measure the densification of SMC samples with both ultrasonic and non-ultrasonic effects in the compaction process. The density profile of the SMC sample with an ultrasonic effect was higher than that with the non-ultrasonic effect. Ultrasonic compaction lowered the internal friction between particles and the die wall, as well as the inter-particle friction inside the die wall. Thanks to this lowered internal friction, particles can rearrange and go into empty spots, making a more compacted SMC sample. In order to verify this high densification phenomenon, the mixture of SMC and lubricant was made with different mixing ratios. As more lubricant was added to the SMC sample with the non-ultrasonic compaction effect, a higher densification was achieved due to the lowered internal friction among the particles themselves. Thus, it was experimentally proved that the ultrasonic compaction effect plays the same role as a lubricant in the compaction process—lowering the internal friction in the system.

With the densification mechanism studied in this paper, a motor made of an SMC core, which was fabricated using an ultrasonic compaction, showed an enhanced efficiency by up to 5% compared to that of a motor made via a conventional method [19,20]. With the proposed mechanism, various applications and modified technologies, such as the example given above, can be achieved and the successful industrialization of ultrasonic compaction in various sectors is expected.

## Figures and Tables

**Table 1:** Variables for ultrasonic compaction.

Parameter	Function	Parameter	Function
u	Vibration amplitude (~20 μm)	*po*	Compacted position
F	Force	*mo*	Modal shape
A	Ultrasonic applied area	*St*	Strength of material
P	Pressure	*Si*	Size of material
t	Operating time (~3 s)	*lu*	Effect of lubricant
f	Frequency (15, 20, 38 Khz)	*Sh*	Shape of material
fr	Friction		

**Table 2:** Coefficient of the relation equation of determining the density of the soft magnetic composites (SMCs).

	a	b	c
SMC550KE *	1.933	0.106	0.894
SMC550 *	−4.830	0.084	1.819
SMC500KE *	3.283	0.125	0.364
SMC500 *	1.266	0.118	0.682

* Trademark of Hoganas, Sweden.
